# Polarimetric Time-Series InSAR for Surface Deformation Monitoring in Mining Area Using Dual-Polarization Data

**DOI:** 10.3390/s25195968

**Published:** 2025-09-25

**Authors:** Xingjun Ju, Sihua Gao, Yongfeng Li

**Affiliations:** 1 School of Emergency Management, China University of Mining and Technology, Xuzhou 221116, China; 2 China Energy Baorixile Energy Co., Ltd., Hulun Buir 021000, China; 3 Research Center for the Transformation and Development of Resource-Based Cities and Rural Revitalization, China University of Mining and Technology, Xuzhou 221116, China

**Keywords:** Time-Series InSAR, dual-polarization Sentinel-1, ground deformation monitoring, Persistent Scatterers, Distributed Scatterers, phase optimization

## Abstract

Timely and reliable surface deformation monitoring is critical for hazard prevention and resource management in mining areas. However, traditional Time-Series Interferometric (TSI) Synthetic Aperture Radar techniques often suffer from low coherent point density in mining environments, limiting their effectiveness. To overcome this limitation, we propose an adaptive Polarimetric TSI (PolTSI) method that exploits dual-polarization Sentinel-1 data to achieve more reliable deformation monitoring in complex mining terrains. The method employs a dual-strategy optimization: amplitude dispersion–based optimization for Permanent Scatterer (PS) pixels and minimum mean square error (MMSE)-based polarimetric filtering followed by coherence maximization for Distributed Scatterer (DS) pixels. Experimental results from an open-pit mining area demonstrate that the proposed approach significantly improves phase quality and spatial coverage. In particular, the number of coherent monitoring points increased from 31,183 with conventional TSI to 465,328 using the proposed approach, corresponding to a 1392% improvement. This substantial enhancement confirms the method’s robustness in extracting deformation signals from low-coherence, heterogeneous mining surfaces. As one of the few studies to apply Polarimetric InSAR (Pol-InSAR) in active mining regions, our work demonstrates the underexplored potential of dual-pol SAR data for improving both the spatial density and reliability of time-series deformation mapping. The results provide a solid technical foundation for large-scale, high-precision surface monitoring in complex mining environments.

## 1. Introduction

Surface deformation monitoring in mining areas is a critical task. Coal mining often triggers a range of surface geohazards, including ground subsidence, surface fissures, and collapses, which can severely damage surrounding resources and the environment, disrupt production, and pose serious threats to human life and property. Therefore, continuous monitoring of surface deformation is essential for ensuring the safety and sustainability of mining operations [1]. Compared with traditional point-based techniques such as leveling [2] and Global Positioning System (GPS) [3], Interferometric Synthetic Aperture Radar (InSAR) [4] utilizes satellite-borne Synthetic Aperture Radar (SAR) imagery to monitor ground deformation. Owing to its advantages—wide spatial coverage, high precision, and low cost—InSAR has been widely applied in various deformation monitoring scenarios [5], including urban subsidence [6,7], infrastructure assessment [8], mining areas [9,10,11,12,13], volcanic activity [14], earthquakes [15], and landslides [16,17].

In recent years, Time-Series InSAR (TSI) [5,18] has emerged as an advanced extension of conventional InSAR techniques. By utilizing multiple SAR acquisitions over the same region, TSI conducts spatiotemporal analysis and error correction of interferometric signals from coherent scatterers, enabling dynamic ground deformation monitoring with centimeter- to millimeter-level accuracy. The TSI methodology was first proposed by Ferretti et al. in 2001 [19,20], introducing the Permanent Scatterer InSAR (PS-InSAR) technique. This approach requires more than 25 SAR scenes to identify high-coherence Persistent Scatterers (PSs) and estimate their time-series deformation. The method was successfully applied to monitor cumulative subsidence over a five-year period in the Ancona region of Italy. Later, in 2004, Hooper et al. extended TSI applications to low-coherence environments by proposing the StaMPS (Stanford Method for Persistent Scatterers) algorithm [21,22]. Assuming linear deformation, the algorithm was tested in the vegetation-covered volcanic area of Eastern California and validated against leveling and GPS data, demonstrating high accuracy and reliability. TSI is particularly effective in urban environments where PS pixels are densely distributed. However, increasing attention has been paid to monitoring Distributed Scatterers (DSs) [4], which are widely present in natural terrains such as rural areas, mining zones, and vegetated regions [20]. DS-InSAR research primarily focuses on two key challenges: the identification of distributed pixels and the optimization of their interferometric phase quality [23]. Zhang et al. [24] applied DS-InSAR to monitor and analyze the Xinjing Open-Pit Mine in Inner Mongolia, China, revealing the spatial evolution and development process of mining-induced geohazards.

Although InSAR has demonstrated high-precision capabilities for surface deformation monitoring and is theoretically capable of large-scale observation in mining areas, its effectiveness in such environments is often compromised. Mining regions are typically characterized by sparse artificial structures and exposed bare soil or desert-like surfaces, which lead to severe signal decorrelation in SAR imagery [25]. Therefore, to ensure the reliability of InSAR-based deformation monitoring in mining areas, improving the phase quality of interferograms is a necessary prerequisite. Traditional filtering and phase optimization techniques [26,27] rely solely on single-polarization SAR data, employing image processing or mathematical models to enhance phase quality. However, they neglect the additional physical scattering information embedded in multi-polarization observations. Since different scatterers respond differently to radar polarization, polarimetric optimization—by leveraging phase information across multiple polarization channels—offers a physically meaningful way to enhance interferometric phase quality, particularly in low-coherence environments such as mining sites.

With the continuous launch of polarimetric SAR (PolSAR) satellites in recent years, the extraction and utilization of phase information from multi-polarized data have become a growing research focus. While traditional InSAR methods primarily rely on single-polarization data, limiting their effectiveness in low-coherence or distributed-scatterer-dominated areas, the availability of multi-polarization observations has enabled more advanced approaches. This advancement has made it feasible to retrieve high-resolution deformation fields through time-series polarimetric InSAR (PolInSAR) processing [28,29], effectively bridging the gap between conventional InSAR techniques and polarimetric vector interferometry.

The concept of polarimetric optimization was first introduced by Cloude and Papathanassiou in 1998 [30], who proposed a general interferometric framework for optimizing coherence using different polarization combinations. Their approach formulates a coherence optimization problem from multi-polarized observations to determine the best scattering mechanism for phase estimation. In 2010, Navarro-Sanchez et al. [31] proposed the Exhaustive Search Polarimetric Optimization (ESPO) method, which identifies the optimal polarization channel by maximizing coherence, thereby improving phase quality. Zahra Sadeghi [32,33] and colleagues applied per-pixel polarimetric optimization to identify the most coherent and dominant scatterers. By integrating this approach with the StaMPS framework, they achieved improved PS selection in non-urban areas, increasing PS density by up to 80% in vegetated regions [34]. By fully exploiting multi-polarization data [35,36,37,38,39,40], these methods have significantly improved both monitoring quality and spatial coverage, opening new possibilities for InSAR applications in complex environments.

Although extensive research has been conducted on polarimetric optimization algorithms, the application of dual-polarization (dual-pol) InSAR techniques for monitoring ground deformation in mining areas remains limited, representing a critical gap in current studies. Most existing approaches rely on a unified optimization strategy, which does not adequately account for the fundamental differences in scattering characteristics between PSs and DSs. To address this limitation, this study focuses on the Baorixile open-pit coal mine in Inner Mongolia as a representative test site, highlighting the novel application of dual-pol Sentinel-1 SAR data in complex mining environments. A total of 48 dual-pol (VV and VH) Sentinel-1 images are utilized to construct a scatterer-type–driven adaptive processing framework. In this framework, PS pixels are optimized using a robust strategy guided by amplitude dispersion, ensuring phase stability and pointwise precision. In contrast, DS regions undergo polarimetric MMSE filtering to enhance interferometric coherence, thereby improving phase quality over spatially homogeneous but low-coherence surfaces. Based on this framework, we propose a polarimetric TSI (PolTSI) method tailored for mining environments, which integrates differentiated polarimetric optimization strategies with TSI processing. This approach effectively improves both the density and the reliability of the monitoring points, particularly in low-coherence mining areas, demonstrating the advantages of dual-pol data to improve deformation monitoring in challenging geological settings.

The structure of this paper is organized as follows: Section 2 presents the polarimetric optimization algorithms employed in this study. Section 3 introduces the study area and dataset. Section 4 describes the experimental results. Section 5 provides a discussion. Finally, Section 6 concludes the study with a summary of the main findings.

## 2. Methods

### 2.1. Polarimetric SAR

In a PolSAR system, the scattering characteristics of radar-received pixel echoes can be represented by the complex scattering matrix *S*, whose each elements represent the complex scattering coefficients under different polarization channels. If the transmitting and receiving antennas are interchangeable and the electromagnetic wave propagates in a medium that satisfies the reciprocity condition, the backscattering matrix should fulfill the reciprocity relation, i.e., $S_{hv}=S_{vh}$ [41,42,43]. Considering the dual-polarization mode of Sentinel-1, which provides only VV (Vertical transmit–Vertical receive) and VH (Vertical transmit–Horizontal receive) channels, the scattering matrix can be simplified as

(1) $$S=\left[\begin{array}{cc} S_{hh} & S_{hv}\\ S_{vh} & S_{vv} \end{array}\right]\Rightarrow S=\left[\begin{array}{cc} 0 & S_{vh}\\ S_{vh} & S_{vv} \end{array}\right]$$

where $S_{vv}$ and $S_{vh}$ are the single-look complex (SLC) SAR images, representing the complex scattering response of the pixels under their respective polarization channels. These images contain both amplitude and phase information, reflecting the scattering characteristics of the pixels. Although this dual-polarization scattering matrix has fewer information dimensions compared to a fully polarimetric system, it offers higher data availability.

To gain deeper insights into the scattering mechanisms of ground objects, PolSAR processing often employs the Pauli basis to represent the complex scattering matrix. The Pauli basis [44] emphasizes the physical characteristics of different scattering types, including surface scattering, double-bounce reflection, and volume scattering. For dual-polarization SAR data (VV and VH channels) provided by Sentinel-1, although the polarization information is limited, a simplified scattering vector can still be constructed under the Pauli basis. Despite the absence of HH channel data, this simplified Pauli vector remains effective in capturing the primary scattering characteristics of terrain features. This capability is particularly valuable in regions where surface scattering (e.g., rocks, soil) and volume scattering (e.g., fractured zones, unconsolidated mineral deposits) exhibit markedly different spatial distributions. Using the Pauli basis, the scattering vector *k* for each polarimetric acquisition can be obtained as shown in (2):

(2) $$k={\left[\begin{array}{c} S_{vv},2S_{vh} \end{array}\right]}^{T}$$

where *T* denotes matrix transpose.

### 2.2. Polarimetric Vector Interferometry

Unlike traditional single-polarization InSAR methods, polarimetric vector interferometry does not rely solely on complex conjugate multiplication of corresponding pixels in primary and secondary images. Instead, it requires simultaneous consideration of the pixels’s polarimetric scattering properties. In single-pol InSAR, the interferometric phase is derived from the complex conjugate multiplication of corresponding pixels in primary image and secondary image. In polarimetric interferometric processing, we first construct polarimetric scattering vectors $k_{1}$ and $k_{2}$ for the primary and secondary images, respectively. We then account for how scattering mechanisms affect coherence. This is accomplished by constructing a Hermitian matrix *T* (the polarimetric interferometric coherence matrix) through an outer product operation, which encodes the polarization coherence information between the two SAR images. This positive semi-definite matrix characterizes the complex coherence across different polarization channel combinations. The scattering vectors from the primary $k_{1}$ and secondary $k_{2}$ images are combined as follows:

(3) $$K={\left[\begin{array}{c} k_{1},k_{2} \end{array}\right]}^{T}$$

The polarimetric interferometric coherence matrix *T* is then constructed by taking the outer product of the combined scattering vector *K* with its own conjugate transpose:

(4) $$T_{4}=K\cdot K^{H}=\left[\begin{array}{cc} T_{11} & \Omega_{12}\\ \Omega_{12}^{H} & T_{22} \end{array}\right]$$

where *H* represent the conjugate transpose operator.

The polarimetric interferometric coherence matrix construction reveals that T comprises four distinct submatrices, each encoding specific polarimetric and interferometric information from the primary/secondary image pair. Specifically, $T_{11}$ and $T_{22}$ are derived from autocorrelation operations (primary or secondary image conjugated with itself), thus containing exclusive polarimetric information for their respective images. Conversely, $\Omega_{12}$ originates from cross-correlation between primary and secondary images, thereby incorporating both polarimetric properties and interferometric phase information. Crucially, temporal decorrelation and other acquisition disturbances cause phase inconsistencies between images, resulting in inherent asymmetry where $\Omega_{12}\neq \Omega_{21}$ reflecting the nonreciprocal nature of complex interferometric phase differences in dual-pol systems.

To further characterize scattering behavior and optimize the interferometric phase, this study introduces a unit complex vector ω, which represents the scattering mechanism of each pixel in terms of the relative amplitudes and phases of its polarimetric components. For dual-polarization SAR data (VV/VH), its mathematical formulation is given by (5)

(5) $$\omega =\left[\begin{array}{c} \cos(\alpha )\\ \sin\left(\alpha \right)e^{j\psi } \end{array}\right],\;\left\{\begin{array}{c} 0\leq \alpha \leq \frac{\pi }{2}\\ -\pi \leq \psi \leq \pi \end{array}\right.$$

where α denotes the scattering angle, characterizing the dominant scattering mechanism and ψ represents the relative phase of the scatterer, encoding its geometric and dielectric properties. The terms *j* (imaginary unit, satisfying $j^{2}=-1$) and *e* (base of natural logarithms) are fundamental mathematical constants governing the complex exponential representation.

To obtain polarimetric vector interferometry results, the polarimetric scattering vectors of the primary and secondary images must be projected onto specific scattering mechanism directions. We let $\omega_{1}$ and $\omega_{2}$ denote the unit complex scattering mechanism vectors for the primary and secondary images, respectively, and let $k_{1}$ and $k_{2}$ represent their corresponding polarimetric scattering vectors. By projecting these scattering vectors onto the scattering mechanism directions, we obtain the corresponding components $u_{1}$ and $u_{2}$, expressed mathematically as

(6) $$u_{1}=\omega_{1}^{H}\cdot k_{1},u_{2}=\omega_{2}^{H}\cdot k_{2}$$

The projected components $u_{1}$ and $u_{2}$ represent the complex scattering responses of the primary and secondary images along the specified scattering mechanism direction, encapsulating both amplitude and phase information of the particular scattering mechanism. Based on these projections, the interferometric phase between the primary and secondary images can be derived through the conjugate product of $u_{1}$ and $u_{2}$:

(7) $$\begin{array}{cc} I & =u_{1}u_{2}^{H}=\omega_{1}^{H}\Omega_{12}\omega_{2} \end{array}$$

The corresponding interferometric phase can be generated as follows:

(8) $$\begin{array}{c} \varphi =arg\left(u_{1}\cdot u_{2}^{H}\right)=arg\left(\omega_{1}^{H}\Omega_{12}\omega_{2}\right) \end{array}$$

### 2.3. Polarimetric Phase Optimization for DSs

DS pixels have strong homogeneity, and their echo signals are composed of coherent superposition of multiple sub-scatterers within the image elements, which are susceptible to spatial and temporal decoherence, resulting in poor phase stability and difficult to be used directly for deformation inversion. Therefore, the key to DS pixel deformation monitoring lies in identifying homogeneous pixels and filtering them to improve the phase quality. In this paper, the HTCI homogeneous pixel identification algorithm proposed by Jiang Mi [45] is first adopted to distinguish PS and DS pixels, and then MMSE polarization filtering is applied to the DS pixels on the basis of which the polarization timing coherence optimization is carried out in order to maximize its phase stability.

MMSE filtering does a good job of preserving image polarization features and edge sharpness [44,46]. This filtering method is based on adaptive weighted average filtering of recognized homogeneous image elements, which is more efficient than the homogeneous filtering used in common DS-InSAR methods.

The MMSE polarimetric filtering process in homogeneous regions begins with computing the polarimetric coherence matrix *T* and its corresponding span image, which represents the total backscattered power:

(9) $$span=Trace\left(T_{11}\right)+Trace\left(T_{22}\right)$$

where Trace() is the trace of the matrix. The filtering weight coefficient *b* is determined based on the span values of statistically homogeneous pixels (SHPs). The specific computation of coef can be found in [44,47], and this coefficient provides a trade-off between noise suppression and feature preservation. In practical applications, 0.51 is empirically set to achieve stable filtering performance.

(10) $$b=\frac{var\left(span_{SHPS}\right)-mean{\left(span_{SHPS}\right)}^{2}coef^{2}}{1+coef^{2}}$$

The MMSE filter is subsequently applied by adaptively weighting the *T* matrices of all SHPs in the neighborhood.

(11) $$T_{filter}=T_{mean}+b\left(T_{ori}-T_{mean}\right)$$

This approach effectively reduces speckle noise while maintaining polarimetric information and spatial resolution, with the trace operation ensuring proper matrix normalization.

### 2.4. Polarimetric Interferometric Phase Optimization

To evaluate the temporal amplitude stability of pixels in SAR time series, dispersion of amplitude ($D_{A}$) was proposed by Ferretti et al. in 2001 [19]. This metric is defined as the ratio between the standard deviation and the mean of the amplitude values across the SAR stack, and it is formulated as

(12) $$D_{A}=\frac{\sigma_{A}}{m_{A}}$$

where $\sigma_{A}$ denotes the standard deviation of the amplitude values and $m_{A}$ is the mean amplitude over the observation period. For time-series polarimetric images, the amplitude dispersion under different scattering mechanisms ω is defined as follows:

(13) $$D_{A}=\frac{1}{\bar{|\omega^{H}k|}}\sqrt{\frac{1}{N}\sum_{i=1}^{N}{\left(|\omega^{H}k|-\bar{|\omega^{H}k|}\right)}^{2}}$$

and

(14) $$\bar{|\omega^{H}k|}=\frac{1}{N}\sum_{i=1}^{N}\left|\omega^{H}k_{i}\right|$$

where *N* denotes the total number of images, the overline indicates the mean value, $k_{i}$ denotes the Pauli basis scattering vector as defined in (2), and ω represents the scattering mechanism vector. This definition ensures a unified scattering mechanism ω across the time-series images, thereby avoiding phase center jumps caused by variations in scattering mechanisms under multi-baseline configurations. Previously, the polarimetric coherence matrix and the scattering mechanism vector were introduced. Analogous to the scalar interferometric coherence coefficient, the polarimetric coherence coefficient is mathematically expressed as

(15) $$\gamma =\frac{|\omega_{1}^{H}\Omega_{12}\omega_{2}|}{\sqrt{\omega_{1}^{H}T_{11}\omega_{1}}\sqrt{\omega_{2}^{H}T_{22}\omega_{2}}}$$

The polarimetric coherence coefficient γ is a complex scalar with a magnitude ranging from 0 to 1. It serves as a quantitative indicator of polarimetric interferometric quality. In (15), the scattering mechanism vectors $\omega_{1}$ and $\omega_{2}$ are complex unit vectors constructed from multiple angular parameters, representing different polarimetric scattering states. Therefore, γ is directly influenced by the choice of scattering mechanisms. By searching for the optimal scattering mechanisms $\omega_{1}$ and $\omega_{2}$ that maximize coherence, the interferometric phase can be projected onto the most coherent polarimetric basis, thereby compensating for decorrelation and improving phase quality. The core objective of polarimetric coherence optimization is to identify the maximum coherence value. In this work, we adopt an Exhaustive Search Polarimetric Optimization (ESPO) algorithm based on the Lagrange multiplier method. First, the Lagrangian function is constructed as follows:

(16) $$L=\omega_{1}^{H}\Omega_{12}\omega_{2}+\lambda_{1}\left(\omega_{1}^{H}T_{11}\omega_{1}-C_{1}\right)+\lambda_{2}\left(\omega_{2}^{H}T_{22}\omega_{2}-C_{2}\right)$$

Here, $\lambda_{1}$ and $\lambda_{2}$ are Lagrange multipliers used to enforce the unit norm constraints on $\omega_{1}$ and $\omega_{2}$. To find the extreme, we set the partial derivatives of *L* with respect to all variables to zero:

(17) $$\frac{\partial L}{\partial \omega_{1}}=\Omega_{12}\omega_{2}+\lambda_{1}T_{11}\omega_{1}=0$$

(18) $$\frac{\partial L}{\partial \omega_{2}}=\Omega_{12}^{H}\omega_{1}+\lambda_{2}T_{22}\omega_{2}=0$$

By setting the eigenvalue as $v=\lambda_{1}\lambda_{2}$, Equations (17) and (18) can be solved to obtain the optimal solution:

(19) $${T_{22}}^{-1}\Omega_{12}^{H}{T_{11}}^{-1}\Omega_{12}\omega_{2}=v\omega_{2}$$

(20) $${T_{11}}^{-1}\Omega_{12}^{H}{T_{22}}^{-1}\Omega_{12}\omega_{2}=v\omega_{1}$$

By solving the system of Equations (17) and (18), two non-negative real eigenvalues can be obtained. The corresponding eigenvectors represent the optimal polarimetric scattering mechanism vectors, denoted as $\omega_{1_{opt}}$ and $\omega_{2_{opt}}$. The optimized interferogram can then be generated using the following expression:

(21) $$u_{1_{opt}}u_{2_{opt}}^{*}=\left(\omega_{1_{opt}}^{H}k_{1}\right){\left(\omega_{2_{opt}}^{H}k_{2}\right)}^{H}=\omega_{1_{opt}}^{H}\Omega_{12}\omega_{2_{opt}}$$

The absolute phases of the two optimal scattering vectors $\omega_{1_{opt}}$ and $\omega_{2_{opt}}$ are not uniquely defined. Therefore, to eliminate phase ambiguities, an additional constraint is introduced as shown in Equation (22). Physically, when there is no temporal decorrelation or scattering-induced decorrelation, i.e., $\omega_{1_{opt}}=\omega_{2_{opt}}$, the constraint is naturally satisfied. However, in practice, due to changes in the polarimetric scattering state between two acquisitions, $\omega_{1_{opt}}\neq \omega_{2_{opt}}$, resulting in phase jumps in the scattering mechanism. To suppress this effect in interferometric phase calculation, it is necessary to fix the scattering mechanism in the secondary image as

(22) $$\arg\left(\omega_{1_{opt}}^{H}\omega_{2_{opt}}\right)=0$$

This ensures consistent phase behavior across the interferometric pair.

For PS pixels, the optimal scattering mechanism ω is defined as the one that minimizes the dispersion of amplitude $D_{A}$. Once the optimal mechanism ω is determined, the polarimetric scattering vector $k_{i}$ of the *i*th image is projected in the direction of ω, yielding the optimized complex pixel value *P* as

(23) $$P=\omega^{H}k_{i}$$

It should be noted that the optimization of $D_{A}$ for PSs is performed based on complex pixel values. In other words, the optimal scattering mechanism ω corresponds to the direction that produces the highest-quality complex signal. Subsequent interferometric processing is then performed using the optimized complex values.

For DS pixels, a polarimetric interferometric coherence matrix is constructed based on single-baseline PolSAR data, and the polarimetric coherence is defined as in Equation (15). A global polarimetric coherence optimization method is proposed, which requires maintaining consistent scattering mechanisms ($\omega_{1}=\omega_{2}$) to avoid phase center shifts; this is known as the principle of the equal scattering mechanism. In multi-temporal and multi-baseline scenarios, one major issue is the potential jump of the scattering phase center. Therefore, it is necessary to maintain $\omega_{1}=\omega_{2}$ to ensure the stability of the phase center in single-baseline polarimetric interferograms. At the same time, it is crucial to unify the scattering mechanism across all temporal interferograms to ensure consistency of scattering behavior over time, thereby avoiding phase inconsistencies. Taking each pixel as an example, the scattering mechanisms for all temporal interferometric pairs are searched, and the corresponding polarimetric coherence values are calculated. The average coherence over time is then determined and the scattering mechanism that maximizes this temporal average is selected as the unique optimal scattering mechanism for that pixel, achieving a unified temporal scattering model [20]. The mathematical expressions are given in Equations (24) and (25):

(24) $$\gamma_{k}\left(\omega \right)=\frac{\omega^{H}\Omega_{12}\omega }{\sqrt{\omega^{H}T_{11}\omega }\sqrt{\omega^{H}T_{22}\omega }}$$

(25) $$|\bar{\gamma }|=\frac{1}{k}\sum_{k=1}^{N^{intf}}\left|\gamma_{k}\left(\omega \right)\right|$$

### 2.5. Time-Series Polarimetric Optimization

**Figure 1:**
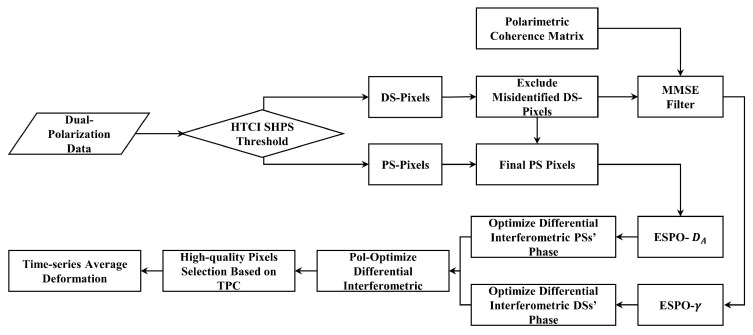
Flowchart of the PolTSI proposed algorithm.

The workflow of time-series polarimetric optimization (as Figure 1) is as follows: First, homogeneous pixel identification is applied to classify the pixels as candidate PS and DS pixels. Among the candidates for DS, pixels with $D_{A}$ less than 0.25 are reclassified as PS, resulting in the final sets of PS and DS pixels. Subsequently, the DS pixels are processed using MMSE polarimetric filtering, while the PS pixels are not filtered in order to preserve their high spatial resolution. For PS pixels, polarimetric optimization is performed by minimizing the $D_{A}$ value, whereas for DS pixels, optimization is performed by maximizing the polarimetric coherence. The final output is a set of polarimetrically optimized interferometric phases that can be used for ground deformation monitoring.

## 3. Study Area and Dataset

**Figure 2:**
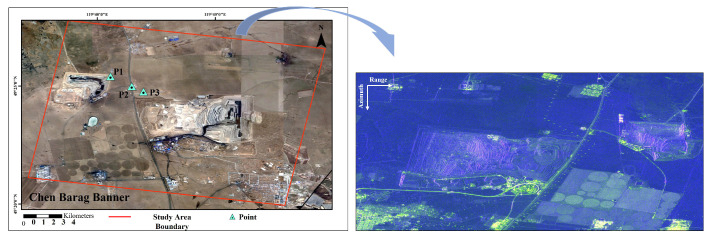
Google Earth optical image of the Baorixile study area. In the left one, the red rectangle delineates the SAR image coverage. The right one shows the RGB composite image (the radar coordinates), where color channels are *R* = $2\left|\bar{VV}\right|$, *G* = $8\left|\bar{VH}\right|$, *B* = $\left|\bar{VV}\right|/4\left|\bar{VH}\right|$.

As shown in Figure 2, the study area is the Baorixile open-pit coal mine, located in Hulunbuir city, Inner Mongolia Autonomous Region, China. The Baorixile open-pit coal mine, one of China’s largest modern coal mines, was selected as the case study due to its representativeness, complex geological setting and significant mining-induced deformation. Since mining began in 1998, continuous excavation has caused widespread ground deformation, including slope instability, stratal displacement, and surface subsidence, forming a typical anthropogenic–geological coupled deformation field. These conditions make Baorixile an ideal site for evaluating the effectiveness of dual-polarization SAR for deformation monitoring. The mining area covers $43.72{\mathrm{km}}^{2}$, with total proven reserves of approximately 1.622 billion tons, an annual production capacity of 35 million tons, and an average stripping ratio of $3.22\;m^{3}$/t, highlighting its status as one of the largest modern open-pit coal mines in China. It lies on the northeast edge of the Hulunbuir High Plain. This region is characterized by typical open-pit mining landscapes and surrounding terrain, where the surface is predominantly covered by natural grasslands and bare soil. Due to the limited presence of man-made structures and strong scatterers, the radar backscattering in this area is dominated by DS pixels. Given the complex scattering mechanisms and low phase stability of DS pixels, advanced polarimetric phase-filtering techniques are required to enhance interferometric coherence and ensure reliable deformation monitoring.

**Figure 3:**
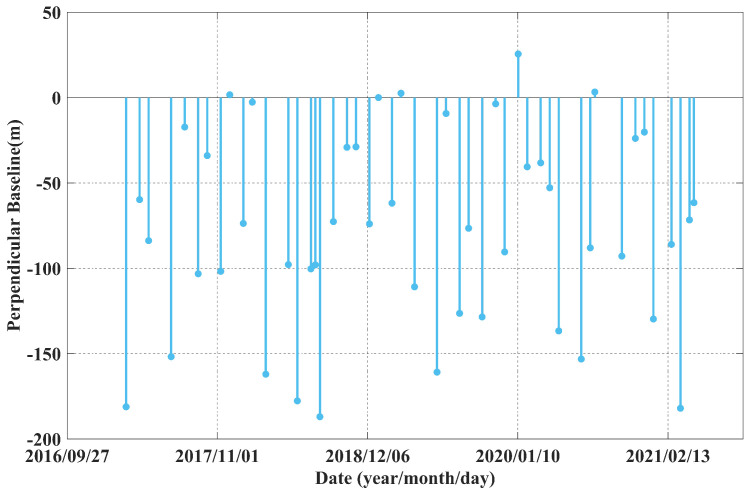
Plot of the temporal and perpendicular baselines of the 47 interferograms generated using the 48 Sentinel-1 images considered in this study.

The radar dataset used in this study consists of dual-polarization C band SAR images acquired by the Sentinel-1 satellite, freely provided by the European Space Agency (ESA). Sentinel-1 data are characterized by excellent orbital stability, a revisit cycle of 12 days (reduced to 6 days in areas jointly covered by Sentinel-1A, Sentinel-1B and Sentinel-1C), and a ground range resolution of approximately 5–20 m depending on the acquisition mode. Combined with their wide spatial coverage, these features make Sentinel-1 data highly suitable for time-series interferometric analysis. A total of 48 SLC images were acquired from March 3, 2017, to April 23, 2021. A single-primary interferometric strategy was employed for time-series analysis, with the primary image dated January 4, 2019. Choosing an image near the middle of the time series as the primary effectively minimizes both temporal and spatial decorrelation, providing a stable reference for interferometric processing. Each SLC scene has a spatial size of 3800 × 900 pixels, covering the main mining area and its surroundings. Using this configuration, a total of 47 interferograms were generated. The distribution of temporal and perpendicular baselines ensures that the interferograms maintain relatively small spatial and temporal separations, as shown in Figure 3.

This TSI study provides a scientific basis and valuable data support for applications such as slope stability assessment, ground subsidence monitoring, and geohazard early warning in open-pit mining areas.

## 4. Results

To assess the effectiveness of the proposed PolTSI technique, a comparative analysis was performed against conventional single-polarization TSI, which relies solely on VV-polarized interferograms. In addition, the optimization capability of PolTSI was systematically evaluated in terms of polarimetric scattering behaviors, aiming to reveal its advantages in enhancing phase quality and deformation retrieval accuracy.

### 4.1. Phase Quality Assessment Based on Temporal Phase Coherence

Phase quality was evaluated using Temporal Phase Coherence (TPC) as a unified selection criterion, which reflects the phase stability for both PSs and optimized DSs. The TPC is calculated using the following equation:

(26) $$\gamma_{TPC}=\frac{1}{N}|\sum_{i=1}^{NN}e^{j\cdot \psi_{noise,i}}|$$

where *N* is the total number of interferograms, *j* is the imaginary unit, and $\psi_{\mathrm{noise},i}$ represents the noise phase component of the *i*th interferogram. The value of $\psi_{\mathrm{noise},i}$ is estimated based on the interferometric phases of neighboring pixels. Considering the surface dynamics of the study area, a threshold of TPC>0.85 was adopted to identify high-quality pixels. Pixels meeting this criterion demonstrate strong temporal phase stability and are ultimately selected as reliable measurement points for deformation monitoring.

**Figure 4:**
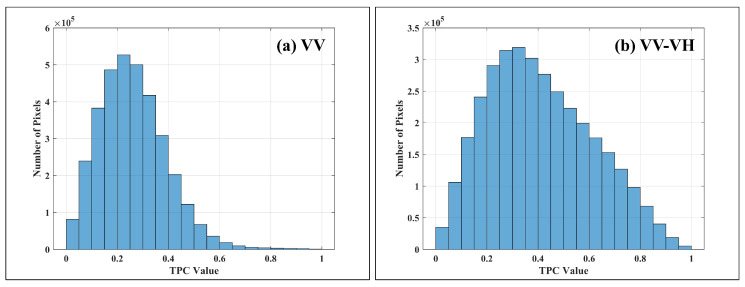
TPC statistics. (**a**) Single-polarization (VV) TPC, (**b**) polarization-optimized TPC results.

The evaluation was carried out using a moving window of size 11×11 to compute TPC for both the original differential interferogram and the optimized interferogram by polarimetric processing. The corresponding TPC maps and their statistical histograms are illustrated for comparative analysis. As shown in Figure 4, the original differential interferogram exhibits relatively poor coherence, with only a sparse distribution of pixels achieving TPC values greater than 0.85. In contrast, after applying the proposed polarimetric optimization method, the number of high-quality pixels with TPC>0.85 increases markedly. This substantial improvement demonstrates that the method effectively enhances phase quality across the scene. Moreover, the overall distribution of TPC values shifts toward higher levels, indicating a significant enhancement in interferometric coherence. These results confirm that polarimetric optimization not only improves the phase stability of DS pixels but also extends the spatial coverage of reliable interferometric measurements, thereby reinforcing the robustness and reliability of time-series deformation monitoring.

### 4.2. Interferogram Processing Results

The differentiated polarimetric optimization strategies were adopted for PS and DS pixels to maximize their respective interferometric advantages. Specifically, for PS points, an amplitude-dispersion-based optimization method was applied to enhance phase stability. For DS points, MMSE polarimetric filtering was first performed to suppress noise, followed by coherence-based optimization to improve phase quality.

**Figure 5:**
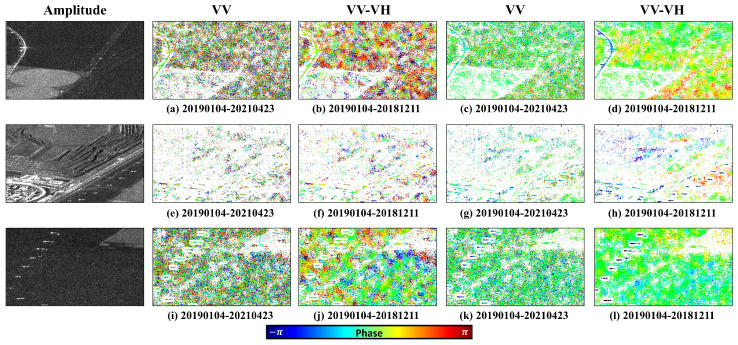
Interferograms of the longest and shortest time baselines.

After standard processing steps, including interferogram generation, flat-Earth phase removal, and topographic phase correction, a total of 47 differential interferograms were produced. To evaluate the effectiveness of the proposed polarimetric optimization, two representative interferometric pairs were selected: one with the shortest temporal baseline (2019-01-04 to 2021-04-23) and one with the longest (2019-01-04 to 2018-12-11), as shown in Figure 5. The results demonstrate a substantial improvement in phase quality, as evidenced by a significant increase in the number of high-quality pixels with TPC>0.85. This improvement was consistent for both short- and long-baseline interferograms, with phase noise greatly reduced compared to the original results.

Specifically, after polarimetric optimization, the speckle noise in homogeneous regions is markedly suppressed, resulting in smoother textures and clearer regional structures. Strong scatterers, such as exposed rocks, retain fine details—including sidelobe signals—which remain clearly visible after filtering. Furthermore, the density of coherent points is significantly increased, allowing subtle surface changes to be more easily detected. These visual enhancements, combined with homogeneous pixel identification and MMSE filtering, clearly demonstrate the effectiveness and adaptability of the proposed approach. Compared with conventional single-polarization processing, the method provides higher phase stability, improved spatial continuity, and greater capability for selecting high-quality monitoring points, thereby establishing a solid foundation for accurate retrieval of ground deformation parameters.

### 4.3. Time-Series Deformation Monitoring Results

**Figure 6:**
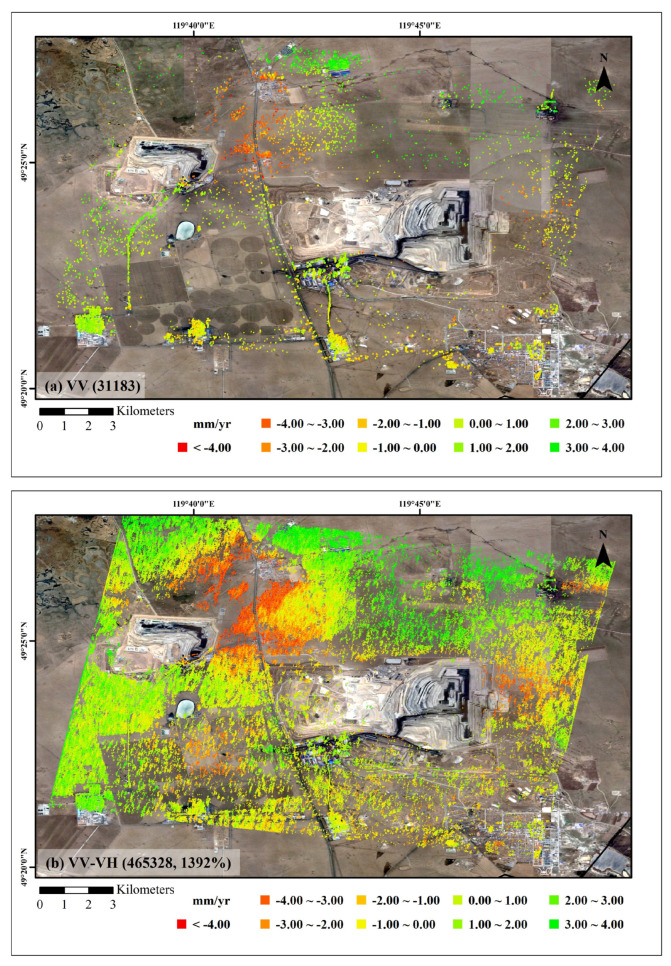
TSI average deformation rate. (**a**) Conventional TSI result, (**b**) PolTSI result.

Surface subsidence monitoring in mining areas has proven to be an effective method for detecting ground deformation. However, conventional PS-InSAR techniques often suffer from a sparse distribution of reliable measurement points in coal mining regions, which significantly limits both the reliability and spatial coverage of the monitoring results. To overcome this limitation, the proposed adaptive polarimetric optimization strategy applies amplitude-dispersion-based optimization to PS points while performing MMSE polarimetric filtering on wide-area DS pixels. This approach retains the resolution of PS points while markedly improving the phase quality of DS points across coalfield regions. Using single-primary TSI processing, the average line-of-sight (LOS) deformation velocity map was generated (Figure 6).

A comparative analysis between conventional TSI and the proposed PolTSI technique shows that, while both methods capture similar large-scale deformation patterns and magnitudes, the PolTSI method provides a dramatic increase in the number and density of coherent measurement points. Specifically, the conventional method yielded only 31,183 points, whereas the PolTSI method detected 465,328, corresponding to a 1392% increase. This substantial improvement not only enhances spatial coverage but also ensures higher reliability for subsequent deformation interpretation. In terms of spatial distribution, the additional coherent points obtained by PolTSI are not randomly scattered but exhibit clear clustering patterns. Most are concentrated in mining-related zones, particularly along pit slopes and adjacent transportation corridors, where significant deformation signals are observed. This spatial consistency reinforces the reliability of the PolTSI-derived results and provides more comprehensive coverage of terrain-dependent deformation features, which are often underrepresented in conventional approaches.

## 5. Discussion

### 5.1. Comparative Consistency of TSI and PolTSI

**Figure 7:**
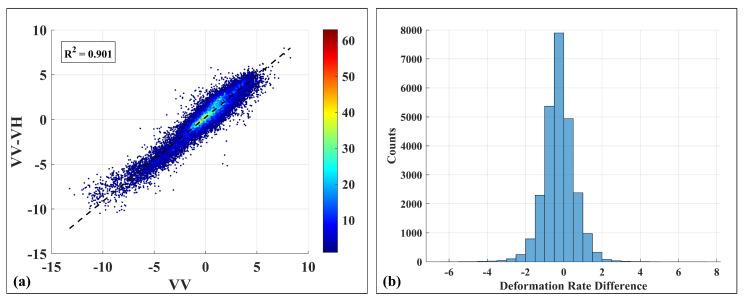
Conventional TSI and PolTSI. (**a**) Correlation graph of deformation rate, (**b**) histogram of deformation rate difference.

To further assess the reliability of both methods in the monitoring of coal mine deformation, a correlation analysis was performed using common high-coherence monitoring points identified by both techniques. A total of 25,556 of these matched points were extracted. The average deformation velocities of these points were plotted in a scatter correlation diagram (Figure 7a), while the velocity differences were statistically analyzed and visualized in a histogram (Figure 7b).

From the correlation analysis in Figure 7a, a strong linear relationship between the two methods is evident, with an R^2^ correlation coefficient of 0.901, indicating high consistency in deformation results for the matched points. The histogram in Figure 7b shows that the velocity differences between matched points are mostly centered around 0 mm/year, with all absolute differences within ±3 mm/year. These findings further validate that both conventional TSI and the proposed PolTSI method provide reliable and mutually consistent deformation monitoring results for mining areas.

**Figure 8:**
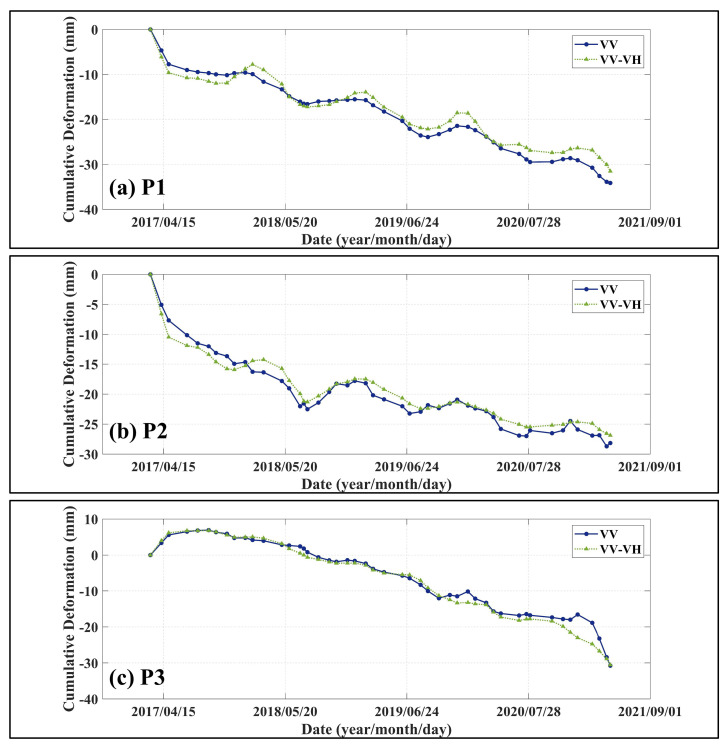
(**a**) P1, (**b**) P2, (**c**) P3 region time sequence deformation diagrams. The dark blue color (dotted) indicates the results of the conventional timing InSAR technique and the light green color (triangles) indicates the results of the planned polarization timing InSAR technique.

To further demonstrate the reliability and effectiveness of the proposed PolTSI monitoring method, three representative target points—P1, P2, and P3—were selected from characteristic areas (as shown in Figure 6). These pixels were consistently detected by both conventional and PolTSI methods. The time-series cumulative deformation curves for these points are illustrated in Figure 8. As observed, both methods yield highly consistent temporal deformation trends for all three pixels. This strong agreement verifies the temporal monitoring capability of the PolTSI technique and further confirms its robustness and accuracy in deformation detection.

### 5.2. Time-Series Monitoring Capability Comparison

**Figure 9:**
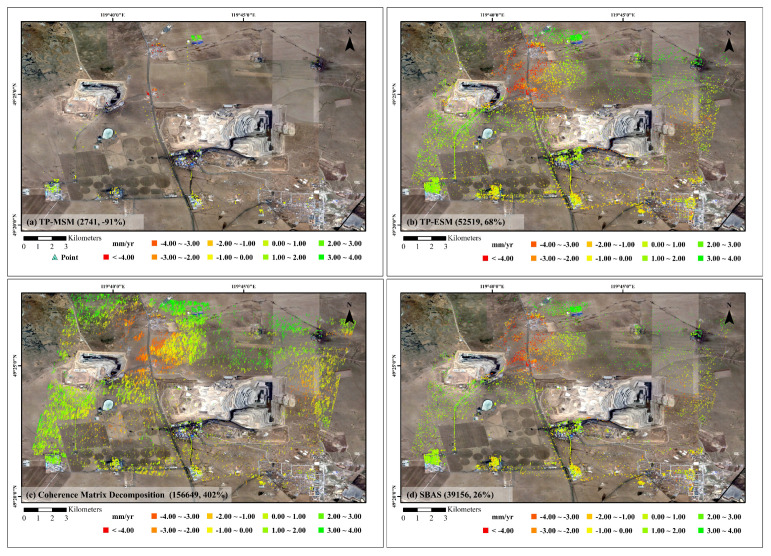
TSI average deformation rate. (**a**) TP-MSM result, (**b**) TP-ESM result, (**c**) coherence matrix decomposition result, (**d**) SBAS-InSAR.

The results presented above clearly demonstrate that the proposed PolTSI technique substantially improves both the quality and density of deformation monitoring in mining areas. The number of reliable measurement points increased from 31,183 with conventional TSI to 465,328 with PolTSI, representing a remarkable 1392% improvement. This is particularly significant given the complex scattering environment and low coherence typically encountered in open-pit coal mining sites, and it validates the effectiveness of the differentiated polarimetric optimization strategies applied to PS and DS pixels. For a more comprehensive evaluation, additional experiments were performed using several widely applied algorithms, including TP-ESM, TP-MSM [35], coherence matrix decomposition [47], and SBAS-InSAR. The results are as follows: TP-ESM produced 52,519 monitoring points (a 68% increase), TP-MSM yielded only 2741 points (a 91% decrease), coherence matrix decomposition achieved 156,649 points (a 402% increase), and SBAS-InSAR showed a 26% increase, as illustrated in Figure 9. These findings indicate that the proposed full-space search algorithm outperforms the other methods in terms of monitoring point density. The relatively lower performance of TP-ESM and TP-MSM can be attributed to their lack of effective phase optimization for DS pixels, while coherence matrix decomposition, although incorporating DS filtering, still yields fewer points than our approach. In terms of computational cost, the proposed algorithm is indeed more intensive due to the exhaustive search for optimal polarimetric scattering vectors. However, this additional processing effort is justified by the substantial improvements in point density, phase stability, and overall data quality. We consider this trade-off reasonable, especially for applications where accurate and dense time-series deformation monitoring is critical.

A significant innovation of this study lies in the dual-strategy optimization: amplitude-dispersion minimization for PS and coherence maximization for DS using MMSE filtering. Unlike conventional approaches that treat all scatterers uniformly, our method adapts to the intrinsic physical characteristics of each scatterer type, leading to more accurate and stable phase retrieval. Furthermore, this study represents one of the few applications of polarimetric InSAR techniques in operational mining environments, a domain where traditional methods often struggle due to sparse point density and signal decorrelation. Our work thus extends the practical applicability of Pol-InSAR and provides a robust methodology for large-scale, high-precision deformation mapping in active mining areas. The improved density of measurement points not only improves spatial coverage but also enables a more detailed analysis of deformation patterns and mining-induced ground instability. This is especially important for early warning systems and risk assessment in resource extraction regions. The integration of polarimetric optimization strategies into TSI workflows offers a scalable solution for long-term monitoring with freely available Sentinel-1 data.

## 6. Conclusions

Ground deformation is a common geohazard in mining regions, directly reflecting subsurface excavation activities and posing significant risks to infrastructure and safety. To overcome the limitations of traditional single-polarization TSI techniques—particularly their sparse distribution of high-coherence pixels in low-scattering or vegetated surfaces—this study proposes an adaptive PolTSI method based on scattering mechanism optimization. By applying differentiated optimization strategies for PS and DS pixels, the proposed approach enhances the interferometric phase quality of DS regions while maintaining the high spatial resolution of PS points. The method was applied to the Baorixile open-pit mining area in Inner Mongolia for large-scale deformation monitoring. Results demonstrate that the polarimetric optimization significantly improves both the quantity and spatial distribution of deformation monitoring points. Specifically, the traditional TSI method yielded only 31,183 monitoring points, whereas the proposed polarimetric InSAR approach achieved 465,328 points—an increase of approximately 1392%. This dramatic improvement directly reflects the method’s effectiveness in extracting reliable deformation signals across heterogeneous scattering environments. Notably, few studies have applied PolTSI techniques in mining areas, making this research one of the first to demonstrate its practical value in complex, deformation-prone settings. The integration of scattering mechanism adaptation with coherence optimization introduces a novel and scalable solution for high-precision deformation mapping in active mining zones. Moreover, this framework has strong potential to support early warning and operational monitoring. By continuously tracking deformation across both mining sites and surrounding regions, it enables timely identification of zones with abnormal displacement, which could indicate potential slope instability or ground failure. Such capabilities provide valuable information for preventive decision-making, risk assessment, and safety management, enhancing the reliability of hazard detection and offering actionable insights for mitigating geohazards before they escalate, thereby contributing to safer and more sustainable mining operations.

## Data Availability

The Sentinel-1A data used in this study are provided by the European Space Agency (ESA).
